# Hospital-based patterns of allergen sensitization among 19,787 patients with suspected allergic diseases in Ningbo, China

**DOI:** 10.3389/fpubh.2026.1786052

**Published:** 2026-04-24

**Authors:** Jiali Tu, Junyuan Li, Qiwei Xie, Cihao Hu, Peng Cheng, Yaowen Wang

**Affiliations:** Department of Otolaryngology Head and Neck Surgery, The First Hospital Affiliated to Ningbo University, Ningbo, China

**Keywords:** allergen sensitization, allergic diseases, China, epidemiology, hospital-based study, Ningbo, specific IgE

## Abstract

**Background:**

Allergic diseases encompass a range of disorders driven by hypersensitive immune responses to environmental allergens, with increasing global prevalence linked to environmental and lifestyle changes. Given regional variations in allergen sensitization patterns, localized epidemiological data are needed to inform clinical management.

**Methods:**

This single-center, hospital-based retrospective study included 19,787 patients with suspected allergic diseases who underwent serum specific IgE (sIgE) testing between January 1, 2019 and December 31, 2021 at the First Hospital Affiliated with Ningbo University in Ningbo, China. Diagnoses were made according to standard clinical criteria, and repeated tests from the same individual were excluded. Sensitization to 15 common inhalant and food allergens was quantitatively assessed using a standardized immunoblot assay (Dixun Biological Genetic Engineering Co., Ltd., China), with sIgE ≥0.35 kU/L defined as positive. We described overall sensitization rates and examined variations across calendar years, sex, age groups, and clinical disease categories.

**Results:**

Dust mites were the most common sensitizing allergens (37.0%), followed by milk (14.3%) and mixed grasses (6.4%). Sensitization rates for several major allergens varied significantly by year. Male patients showed higher sensitization rates to multiple allergens than females. Age-stratified analysis revealed a shift from predominant food allergen sensitization (e.g., milk, egg white) in early childhood to inhalant allergen sensitization (e.g., house dust mites, mixed grasses) in adolescence and adulthood. Sensitization patterns also differed significantly across clinical disease entities, with distinct profiles observed in patients with allergic rhinitis, asthma, and atopic dermatitis.

**Conclusion:**

This large hospital-based study delineates the allergen sensitization profile among patients with suspected allergic diseases in Ningbo, eastern China. These findings underscore the importance of considering age, sex, year of testing, and disease type when interpreting sIgE results in clinical practice. Because sIgE positivity reflects sensitization rather than confirmed clinical allergy, our results should be interpreted as sensitization patterns in a symptomatic, selected population rather than as prevalence estimates in the general population. Nonetheless, establishing a regionally relevant allergen sensitization spectrum may help optimize diagnostic panels, inform targeted prevention strategies, and support precision management of allergic diseases in this region.

## 1 Introduction

Allergic diseases are a group of disorders characterized by inappropriate immune responses to otherwise harmless environmental substances, often involving the production of allergen-specific immunoglobulin E (sIgE) antibodies (1). These conditions have a strong hereditary component and encompass a wide range of clinical manifestations, predominantly affecting the respiratory tract, skin, and eyes (2). In recent decades, both the prevalence and incidence of allergic diseases have increased worldwide, partly due to factors such as air pollution, climate change, and loss of biodiversity, placing a substantial burden on health and quality of life (3).

Patterns of allergen sensitization vary considerably by geographical region, climate, environmental exposures, and individual lifestyle factors (4). Therefore, region-specific data on allergen sensitization are essential to guide clinical diagnosis, interpret sIgE test results, and develop targeted prevention strategies.

Ningbo is a coastal city in Eastern China with a humid subtropical monsoon climate (5), where environmental conditions, aeroallergen exposure, and dietary habits may differ from those in inland or northern regions (4, 6). However, large-scale hospital-based data on allergen sensitization patterns in this area remain limited.

In this retrospective hospital-based study, we analyzed serum sIgE test results from 19,787 patients with suspected allergic diseases who attended the First Hospital Affiliated with Ningbo University between January 1, 2019 and December 31, 2021. Our aim was to characterize allergen sensitization patterns across different age groups, sexes, calendar years, and clinical disease categories (including allergic rhinitis, chronic rhinosinusitis, asthma, urticaria, atopic dermatitis, allergic dermatitis, and allergic conjunctivitis), so as to provide regionally relevant evidence for clinical practice in Ningbo. Throughout this study, we use the term “sensitization” to refer to sIgE positivity, recognizing that it does not necessarily equate to clinically manifest allergy.

## 2 Methods

### 2.1 Study population

This retrospective hospital-based study included patients who visited the First Hospital Affiliated with Ningbo University (Ningbo, China) between January 1, 2019 and December 31, 2021 and were referred for serum allergen-specific IgE (sIgE) testing due to suspected allergic diseases. All patients who met these criteria and underwent sIgE testing during the study period were consecutively included.

All patients had at least one physician-diagnosed allergic condition, including allergic rhinitis (AR), chronic rhinosinusitis (CRS) with a suspected allergic component, asthma, urticaria, atopic dermatitis (AD), allergic dermatitis, and allergic conjunctivitis (AC). Allergic diseases were diagnosed by board-certified clinicians in routine practice at the First Hospital Affiliated with Ningbo University. Diagnoses were based on the patients’ clinical history, physical examination, and ancillary tests (such as lung function, imaging, and routine laboratory investigations) according to the treating physician’s judgment, and were generally consistent with contemporary national or international guideline recommendations (e.g., for allergic rhinitis, asthma, chronic rhinosinusitis, and atopic dermatitis), although formal guideline checklists were not systematically recorded in the electronic medical records. When patients had multiple allergic diagnoses, the primary diagnosis recorded at the index visit was used to classify them into one disease category for subgroup analyses; comorbidities were described descriptively and considered in the interpretation of results. Information on CRS phenotype (with vs. without nasal polyps) was not systematically recorded, so we were unable to stratify CRS by polyp status, which is known to influence atopy prevalence.

Patients with documented parasitic infections, autoimmune diseases, primary or secondary immunodeficiencies, or other conditions known to substantially affect IgE production were excluded. If a patient underwent more than one sIgE test during the study period, only the first test result was included in the analysis to avoid duplication.

After applying these criteria, a total of 19,787 unique patients were included, comprising 8,604 males and 11,183 females (Table 1). Their ages ranged from 2 to 117 years, with a mean age of 33.9 years.

**Table 1:** Characteristics of participants.

Groups	Number of subjects	Percentage (%)
Year group		
2019	7,017	35.5
2021	5,899	29.8
2020	6,871	34.7
Sex		
Male	8,604	43.5
Female	11,183	56.4
Age		
>2–5 years	338	1.7
>6–13 years	3,324	16.8
>14–17 years	900	4.5
>18–35 years	6,510	32.9
>36–60 years	6,532	33.0
≥61 years	2,182	11.0
Diagnosis		
Allergic rhinitis	9,617	48.6
Chronic rhinosinusitis	2,790	14.1
Asthma	1,583	8.0
Urticaria	3,997	20.2
Atopic dermatitis	514	2.6
Allergic dermatitis	949	4.8
Atopic conjunctivitis	337	1.7

The study protocol was reviewed and approved by the Ethics Committee of the First Hospital Affiliated with Ningbo University (approval number: 181RS). Because of the retrospective design and use of anonymized laboratory data, the requirement for written informed consent was waived.

### 2.2 Detection methods

Peripheral venous blood (5 mL) was collected from each patient and allowed to clot at room temperature for approximately 30 min. Serum was then separated by centrifugation at 3,500 rpm for 10 min. Serum sIgE levels were quantitatively measured using a standardized immunoblot assay (DX-Blot 45II fully automatic immunoblotting instrument, Dixun Biological Genetic Engineering Co., Ltd., Hangzhou, China), following the manufacturer’s instructions.

The test panel included common inhalant and food allergens. Inhalant allergens comprised short ragweed, mugwort (*Artemisia absinthium*), dog dander, mixed grass, cat dander, the fungal allergen mix, tree pollen mix, dust mites, and cockroach. Food allergens included peanut, egg white, lobster and scallop mix, milk, beef, and crab. The fungal allergen mix contained *Alternaria alternata*, Aspergillus fumigatus, *Cladosporium herbarum*, and Penicillium notatum. All sIgE results were reported in kU/L.

We defined sensitization to a given allergen as an sIgE concentration of 0.35 kU/L or higher, with values below this threshold considered negative. This binary classification (sensitized vs. not sensitized) was used in all subsequent analyses. Throughout the manuscript, “sensitization” refers strictly to sIgE positivity and does not imply clinically confirmed allergy.

### 2.3 Statistical analyses

Serum allergen test results for the 19,787 patients were treated as categorical variables (sensitized vs. not sensitized) and are reported as frequencies and percentages. We compared baseline characteristics and sensitization rates between groups (e.g., by sex, age group, clinical disease category, and year) using the chi-square test.

To further explore factors associated with sensitization, we built separate multivariable logistic regression models for seven major allergens that had a positive rate above 3% and were clinically relevant in the study region: short ragweed, mixed grass pollen, cat dander, house dust mites, peanut, egg white, and milk. In each model, sensitization to the specific allergen (yes/no) served as the dependent variable, while age, sex, and clinical disease category (AR, CRS, asthma, urticaria, AD, allergic dermatitis, and AC) were included as independent variables. Age was entered either as a continuous variable (per 10-year increment) or as a categorical variable, depending on whether the relationship was non-linear. For allergens where the age-squared term was not significant in preliminary analyses (short ragweed, peanut, and house dust mites), we modeled age as a continuous variable per 10-year increase. For allergens with a significant age-squared term (mixed grass pollen, egg white, cat dander, and milk), we categorized age into six groups: 2–5, 6–13, 14–17, 18–35 (reference), 36–60, and ≥61 years. Adjusted odds ratios (aORs) and 95% confidence intervals (CIs) were derived from these models.

We also formally assessed age-related trends in sensitization. For short ragweed, peanut, and dust mites, we quantified linear age effects using the aOR per 10-year increase in age. For mixed grass pollen, egg white, cat dander, and milk, we estimated age-specific odds ratios for predefined age groups (with 18–35 years as the reference) and calculated *p* values for linear trend across age categories.

To evaluate whether sex modified these associations, we introduced age-by-sex interaction terms into the logistic regression models for each allergen. When a statistically significant interaction (*P* for interaction <0.05) was detected, we fitted separate sex-stratified models to estimate sex-specific age effects.

All statistical analyses were performed using SPSS version 26.0 (IBM Corp., Armonk, NY, United States). A two-tailed *p* < 0.05 was considered statistically significant. For multiple comparisons across allergens and subgroups, Benjamini–Hochberg false discovery rate control was applied as appropriate.

## 3 Results

### 3.1 Overall profiles of allergen sensitization

Table 2 presents the sensitization rates for the 15 tested allergens over the three-year study period. Dust mites were the most common, with 7,317 positive tests out of 19,787 patients (37.0%). At the other end, mugwort had the lowest rate, at just 1.2% (230 cases). Among inhalant allergens, dust mites (37.0%, 7,317/19,787), mixed grass (6.4%, 1,268/19,787), and cat dander (4.2%, 832/19,787) ranked highest. For food allergens, milk was the most frequently detected (14.3%, 2,831/19,787), followed by egg white (5.5%, 1,084/19,787) and peanut (4.0%, 787/19,787).

**Table 2:** Sensitization rates of different allergen species [n (%)].

Groups	Allergen species	Number(%)
Inhalant allergens	Ragweed	660(3.3)
	Mugwort	230(1.2)
	Dog dander	321(1.6)
	Mixed grass	1,268(6.4)
	Cat dander	832(4.2)
	Fungal allergen mix	295(1.50)
	Tree pollen mix	431(2.2)
	Dust mites	7,317(37.0)
	Cockroaches	416(2.1)
Food allergens	Peanuts	787(4.0)
	Egg white	1,084(5.5)
	Lobster and scallop mix	525(2.7)
	Milk	2,831(14.3)
	Beef	289(1.5)
	Crab	552(2.8)

### 3.2 Sensitization profiles by year

Over the study period (January 1, 2019 to December 31, 2021), sensitization rates for mugwort and milk remained stable across the 3 years (*p* > 0.05) (Table 3). For the other 13 allergens, rates varied significantly by year (all *p* < 0.05) (Table 3), pointing to shifts in sensitization patterns among hospital-tested patients over time. We revisit these year-to-year variations in the Discussion, considering possible changes in environmental exposures and patient composition.

**Table 3:** Sensitization rates of allergen species in year [n(%)].

Allergen species	2019	2020	2021	χ^2^
Ragweed	225(3.2)	172(2.9)	263(3.8)	8.8*
Mugwort	89(1.3)	55(0.9)	86(1.3)	3.9
Dog dander	81(1.2)	48(0.8)	192(2.8)	92.9***
Peanuts	353(5.0)	290(4.9)	144(2.1)	97.7***
Mixed grass	417(5.9)	336(5.7)	515(7.5)	21.1***
Egg white	526(7.5)	261(4.4)	297(4.3)	85.6***
Lobster and scallop mix	239(3.4)	143(2.4)	143(2.1)	25.3***
Cat dander	255(3.6)	232(3.9)	345(5.0)	18.1***
Fungal allergen mix	99(1.4)	61(1.0)	135(2.0)	19.2***
Milk	1,016(14.5)	795(13.5)	1,020(14.9)	5.1
Beef	163(2.3)	45(0.8)	81(1.2)	60.0***
Tree pollen mix	106(1.5)	72(1.2)	253(3.7)	113.0***
Dust mites	2,275(32.4)	2,177(36.9)	2,865(41.7)	128.2***
Crab	196(2.8)	136(2.3)	220(3.2)	9.4**
Cockroaches	100(1.4)	64(1.1)	252(3.7)	127.1***
Over all sensitization rates	3,686(52.5)	3,601(52.4)	3,863(56.2)	26.1***

*p < 0.05, **p < 0.01, ***p < 0.001. Bold values indicate that the chi‑square values are statistically significant.

### 3.3 Sensitization profiles by sex

**Figure 1:**
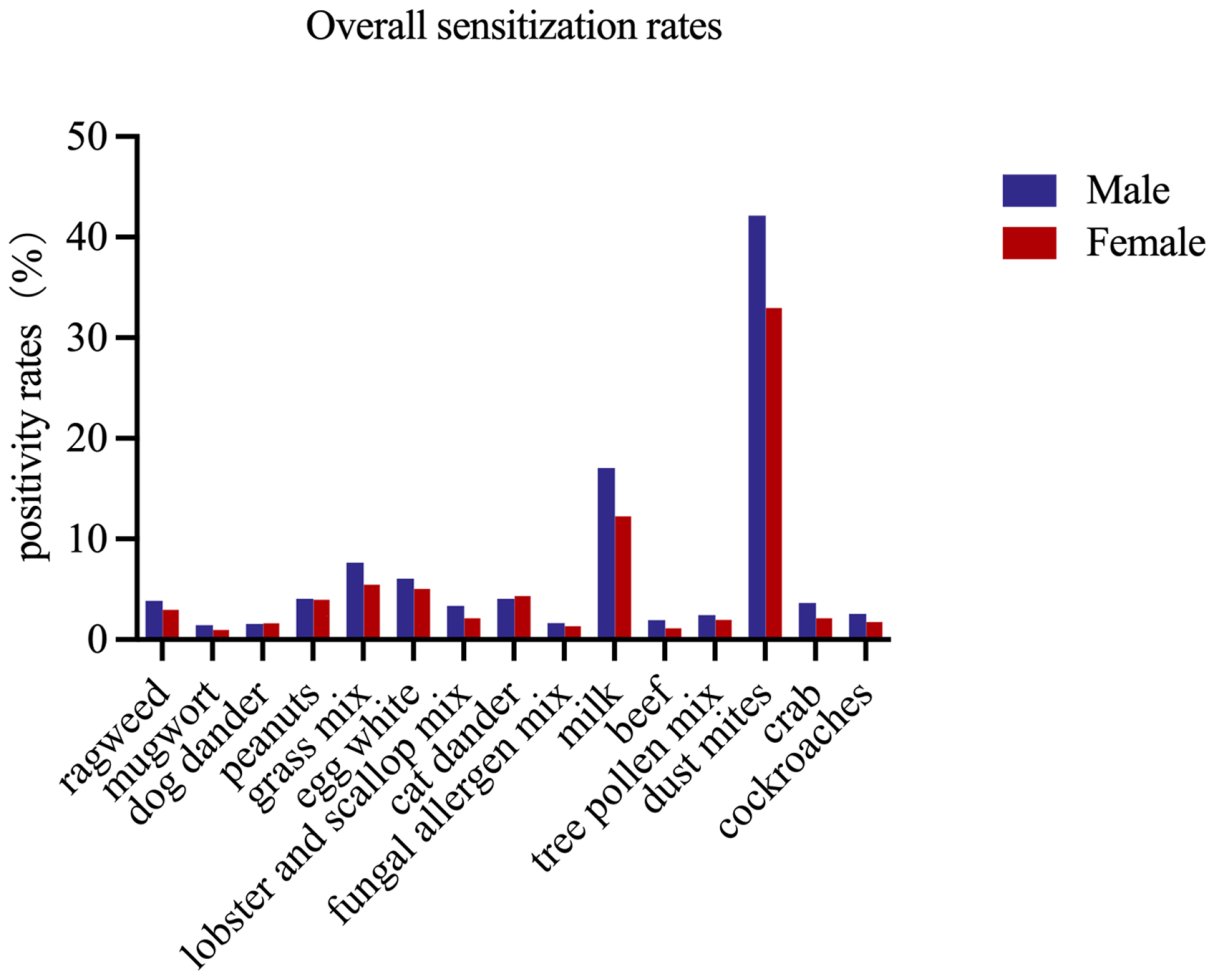
Prevalence of sensitization to allergen species in sex.

**Figure 2:**
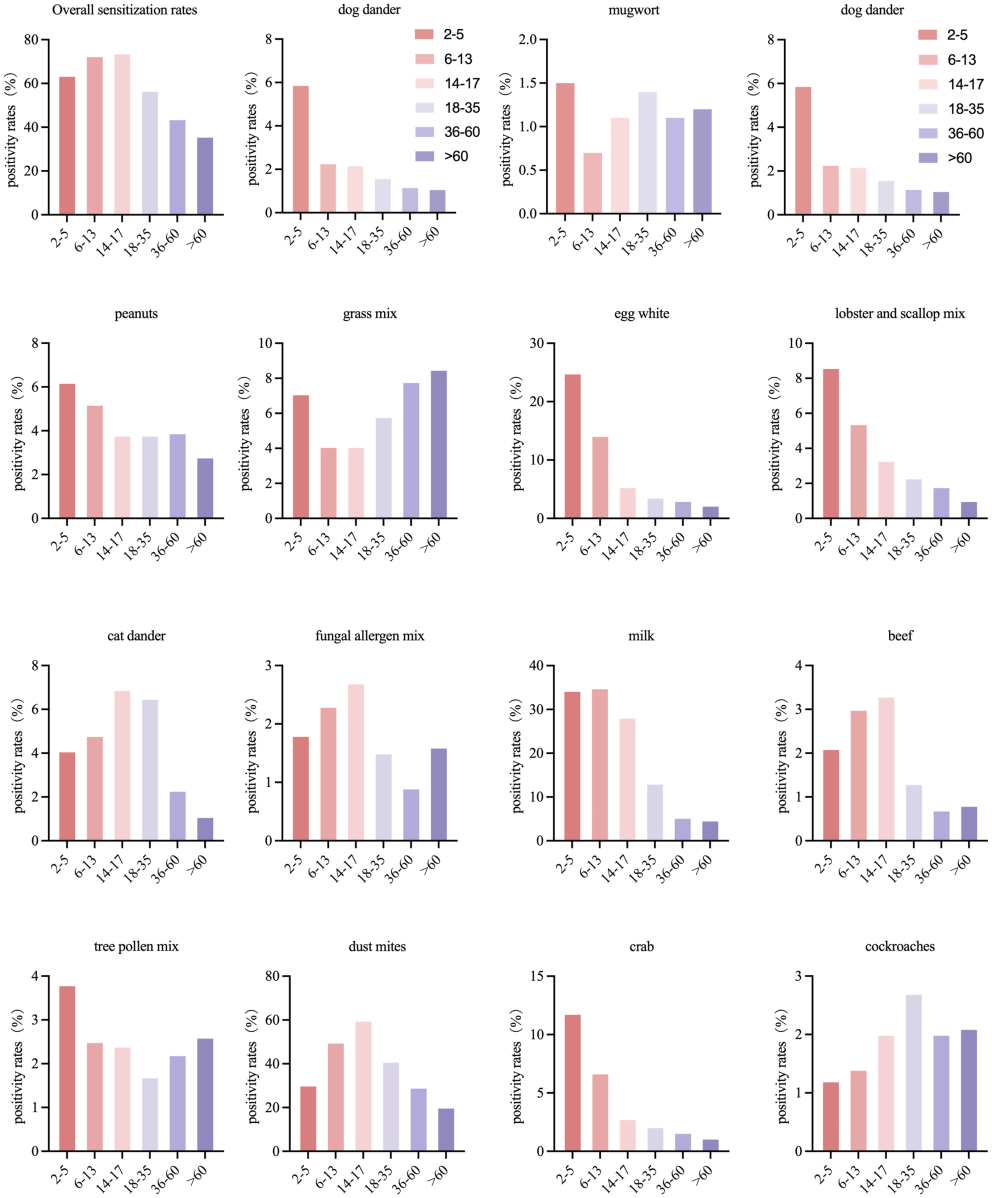
Prevalence of sensitization to allergen species in age.

Of the 19,787 patients, 8,604 (43.5%) were male and 11,183 (56.5%) were female. Sensitization rates for dog dander, peanut, cat dander, the fungal allergen mix, and tree pollen mix were comparable between sexes (all *p* > 0.05) (Table 4). For the other 10 allergens, rates were higher in males than in females (all *p* < 0.05) (Table 4; Figure 1). Dust mites, milk, and mixed grass were the three most common allergens in both sexes. Multivariable logistic regression confirmed these patterns: males had higher adjusted odds of sensitization to several inhalant allergens and to milk, while females had higher odds for cat dander (Supplementary Tables S1, S2; Figure 2).

**Table 4:** Sensitization rates of allergen species in sex [n(%)].

Allergen species	Male	Female	χ^2^
Ragweed	328(3.8)	332(3.0)	10.7**
Mugwort	119(1.4)	111(1.0)	6.5*
Dog dander	133(1.5)	188(1.7)	0.6
Peanuts	342(4.0)	445(4.0)	0.0
Mixed grass	656(7.6)	612(5.5)	37.5***
Egg white	518(6.0)	566(5.1)	8.6**
Lobster and scallop mix	282(3.3)	243(2.2)	22.9***
Cat dander	341(4.0)	491(4.4)	2.2
Fungal allergen mix	139(1.6)	156(1.4)	1.6
Milk	1,460(17.0)	1,371(12.3)	87.9***
Beef	160(1.9)	129(1.2)	16.8***
Tree pollen mix	206(2.4)	225(2.0)	3.3
Dust mites	3,623(42.1)	3,694(33.0)	171.9***
Crab	307(3.6)	245(2.2)	34.0***
Cockroaches	211(2.5)	205(1.8)	9.1**
Over all sensitization rate	5,018(58.3)	5,613(50.2)	129.7***

*p < 0.05, **p < 0.01, ***p < 0.001. Bold values indicate that the chi‑square values are statistically significant.

### 3.4 Sensitization profiles by age

We divided the cohort into six age groups: preschool (2–5 years, *n* = 338), school-age (6–13 years, *n* = 3,324), adolescent (14–17 years, *n* = 900), young adult (18–35 years, *n* = 6,510), middle-aged (36–60 years, *n* = 6,532), and older adult (>60 years, *n* = 2,182). One patient was excluded due to missing age data. Sensitization to mugwort (*Artemisia absinthium*) did not vary significantly across age groups (*p* > 0.05) (Table 5), whereas the other 14 allergens showed clear age-related differences (all *p* < 0.05) (Table 5).

**Table 5:** Sensitization rates of allergen species in age [n(%)].

Allergen species	2–5	6–13	14–17	18–35	35–60	>61	χ^2^
Ragweed	13 (3.8)	91 (2.7)	25 (2.8)	197 (3.0)	233 (3.6)	101 (4.6)	19.2**
Mugwort	5 (1.5)	23 (0.7)	10 (1.1)	91 (1.4)	74 (1.1)	27 (1.2)	10.9
Dog dander	20 (5.9)	78 (2.3)	20 (2.2)	104 (1.6)	76 (1.2)	23 (1.1)	65.1***
Peanuts	21 (6.2)	172 (5.2)	34 (3.8)	245 (3.8)	254 (3.9)	61 (2.8)	25.9***
Mixed grass	24 (7.1)	135 (4.1)	37 (4.1)	376 (5.8)	510 (7.8)	186 (8.5)	80.7***
Egg white	84 (24.9)	473 (14.2)	49 (5.4)	233 (3.6)	197 (3.0)	48 (2.2)	903.7***
Lobster and scallop mix	29 (8.6)	179 (5.4)	30 (3.3)	147 (2.3)	119 (1.8)	21 (1.0)	189.2***
Cat dander	14 (4.1)	160 (4.8)	62 (6.9)	421 (6.5)	150 (2.3)	25 (1.1)	211.6***
Fungal allergen mix	6 (1.8)	75 (2.3)	24 (2.7)	99 (1.5)	57 (0.9)	34 (1.6)	39.0***
Milk	116 (34.3)	1,160 (34.9)	254 (28.2)	850 (13.1)	348 (5.3)	103 (4.7)	2003.4***
Beef	7 (2.1)	99 (3.0)	30 (3.3)	87 (1.3)	48 (0.7)	18 (0.8)	106.7***
Tree pollen mix	13 (3.8)	83 (2.5)	22 (2.4)	110 (1.7)	146 (2.2)	57 (2.6)	15.6**
Dust mites	102 (30.2)	1,656 (49.8)	538 (59.8)	2,674 (41.1)	1,909 (29.2)	438 (20.1)	925.6***
Crab	40 (11.8)	223 (6.7)	25 (2.8)	134 (2.1)	106 (1.6)	24 (1.1)	358.8***
Cockroaches	4 (1.2)	45 (1.4)	18 (2.0)	176 (2.7)	128 (2.0)	45 (2.1)	22.6***
Over all sensitizatione rate	215 (63.6)	2,413 (72.6)	665 (73.9)	3,697 (56.8)	2,862 (43.8)	779 (35.7)	1,204.2***

*p < 0.05, **p < 0.01, ***p < 0.001. Bold values indicate that the chi‑square values are statistically significant.

In preschool children, the most common allergens were milk, dust mites, and egg white, a pattern that persisted in school-age children. Among adolescents and young adults, dust mites, milk, and cat dander formed the top three. For middle-aged and older adult patients, dust mites, mixed grass, and milk ranked highest. Multivariable logistic regression and trend analyses further clarified these age-related patterns: sensitization to milk, egg white, cat dander, and house dust mites declined with age, while sensitization to mixed grass pollen and short ragweed increased in older groups (Supplementary Tables S1, S2; Figure 2).

### 3.5 Sensitization profiles by clinical disease category

Based on the clinical diagnosis at consultation, we classified allergic diseases into seven categories: AR (9,617 cases), CRS (2,790 cases), asthma (1,583 cases), urticaria (3,997 cases), AD (514 cases), allergic dermatitis (949 cases), and AC (337 cases).

Sensitization rates for ragweed, mugwort, the fungal allergen mix, and beef did not vary significantly across disease groups (all *p* > 0.05) (Table 6). For the other 11 allergens, rates differed by disease category (all *p* < 0.05) (Table 6), pointing to disease-specific patterns.

**Table 6:** Sensitization rates of allergen species in clinical disease category [n(%)].

Allergen species	AR	CRS	asthma	urticaria	AD	allergic dermatitis	AC	χ^2^
Ragweed	206 (3.0)	74 (2.8)	51 (3.9)	94 (3.8)	13 (3.9)	21 (3.4)	5 (3.3)	8.7
Mugwort	71 (1.0)	24 (0.9)	19 (1.4)	26 (1.0)	2 (0.6)	7 (1.1)	3 (2.0)	4.6
Dog dander	121 (1.8)	22 (0.8)	35 (2.6)	23 (0.9)	6 (1.8)	7 (1.1)	5 (3.3)	33.9***
Peanuts	309 (4.5)	112 (4.2)	43 (3.2)	84 (3.4)	26 (7.8)	26 (4.3)	6 (3.9)	20.2**
Mixed grass	378 (5.5)	146 (5.5)	94 (7.1)	169 (6.8)	27 (8.1)	41 (6.7)	5 (3.3)	17.3*
Egg white	376 (5.5)	139 (5.2)	50 (3.8)	92 (3.7)	38 (11.4)	31 (5.1)	11 (7.2)	46.1***
Lobster and scallop mix	187 (2.7)	68 (2.5)	32 (2.4)	52 (2.1)	25 (7.5)	13 (2.1)	5 (3.3)	26.0***
Cat dander	355 (5.2)	86 (3.2)	78 (5.9)	68 (2.7)	15 (4.5)	13 (2.1)	14 (9.2)	62.0***
Fungal allergen mix	96 (1.4)	42 (1.6)	21 (1.6)	29 (1.2)	11 (3.3)	9 (1.5)	2 (1.3)	**8.9**
Milk	1,060 (15.5)	348 (13.0)	100 (7.6)	305 (12.3)	75 (22.6)	69 (11.3)	32 (21.1)	104.4***
Beef	102 (1.5)	39 (1.5)	14 (1.1)	35 (1.4)	8 (2.4)	14 (2.3)	4 (2.6)	**8.1**
Tree pollen mix	103 (1.5)	29 (1.1)	27 (2.0)	54 (2.2)	13 (3.9)	16 (2.6)	7 (4.6)	31.2***
Dust mites	3,160 (46.1)	994 (37.2)	499 (37.7)	810 (32.7)	156 (47.0)	160 (26.2)	72 (47.4)	283.8***
Crab	200 (2.9)	60 (2.2)	40 (3.0)	46 (1.9)	27 (8.1)	11 (1.8)	6 (3.9)	40.2***
Cockroaches	108 (1.6)	31 (1.2)	33 (2.5)	77 (3.1)	9 (2.7)	19 (3.1)	1 (0.7)	42.0***
Over all sensitization rate	4,153 (60.6)	1,421 (53.2)	684 (51.7)	1,236 (49.9)	215 (64.8)	278 (45.5)	100 (65.8)	158.1***

*p < 0.05, **p < 0.01, ***p < 0.001. Bold values indicate that the chi‑square values are statistically significant.

Among patients with AR, dust mites were the most common sensitization (42.1%), followed by milk (18.3%); mixed grass and egg white tied for third (5.5% each). In the CRS, asthma, urticaria, and allergic dermatitis groups, the top three allergens were dust mites, milk, and mixed grass. For AD, dust mites, milk, and egg white ranked highest. AC showed a different profile, with dust mites, milk, and cat dander as the most frequent sensitizations.

We further examined these patterns using multivariable logistic regression adjusted for age and sex. Associations between individual diseases and specific allergens were generally modest and often weaker than the effects of age and sex, though some links—such as urticaria with egg white and allergic dermatitis with dust mites—remained statistically significant (Supplementary Table S1).

## 4 Discussion

Allergic diseases are on the rise worldwide and pose a considerable public health burden (7). Interpreting diagnostic tests and planning preventive strategies calls for region-specific data on allergen sensitization (8). In this large hospital-based cohort from Ningbo, eastern China, we characterized sensitization to 15 common inhalant and food allergens among 19,787 patients with suspected allergic diseases, and examined how sensitization varied by age, sex, year, and clinical phenotype.

Overall, 53.7% of patients were sensitized to at least one allergen. Sensitization rates ranged from 1.16 to 36.98% for inhalant allergens and from 1.46 to 14.31% for food allergens, with inhalant sensitization—particularly to house dust mites—being more common. These figures reflect sensitization patterns in a symptomatic referred population and should not be taken as estimates of allergy prevalence in the general population.

### 4.1 Dust mites in a humid coastal environment

Dust mites were the most common sensitizing allergens across nearly all age groups and disease categories, consistent with findings from other regions in China and elsewhere (4, 9–11). Ningbo’s warm, humid subtropical monsoon climate, combined with widespread use of air conditioning, likely creates favorable conditions for mite proliferation and sustained indoor exposure. In our age-adjusted analyses, mite sensitization decreased significantly with age, suggesting that younger patients bear the highest burden. This pattern may be explained by a combination of factors: intense indoor exposure early in life, subsequent development of partial tolerance, changes in living conditions over time, or age-related shifts in immune function.

### 4.2 Food sensitization in early life and the atopic march

Milk and egg white topped the list of food sensitizations, especially in preschool and school-age children, and their rates dropped sharply with age. Multivariable models confirmed that the odds of sensitization to both were highest in early childhood and substantially lower in adults, echoing findings from previous studies in China and Europe (12). This aligns with the established pattern of early-onset food sensitization (8, 13, 14): Diets in early infancy are predominantly milk-based, followed by the introduction of egg and other complementary foods at a time when the intestinal barrier and immune system are still maturing (15, 16). During this period, increased intestinal permeability and incomplete enzymatic digestion may allow intact proteins to pass through and promote sensitization; as gastric acidity, digestive enzymes, and mucosal immune regulation mature with age, food sensitization often subsides (17, 18).

The age-specific trajectories we observed—early peaks for food sensitization followed by a later predominance of respiratory sensitization to mites and pollens—are broadly consistent with the concept of the “atopic march,” in which food sensitization in early life precedes subsequent airway sensitization and allergic respiratory disease (19).

Of note, this retrospective study did not systematically collect data on immediate symptoms after food ingestion or perform oral food challenges. Food-specific sIgE positivity therefore reflects sensitization rather than confirmed food allergy. Accordingly, we describe “sensitization to milk/egg white” and avoid terms such as “milk allergy.”

### 4.3 Herbaceous pollen and aeroallergen patterns in older age

Mixed grass was the most common herbaceous pollen sensitization in this cohort. Sensitization to mixed grass pollen and short ragweed increased with age in both descriptive and regression analyses, particularly among middle-aged and older adults. This pattern differs from the childhood-limited trajectory of milk and egg white sensitization, and suggests that cumulative aeroallergen exposure and age-related changes in mucosal immunity play a greater role for seasonal pollens (20). Our findings are consistent with the age-stratified sensitization dynamics reported by Giovanni et al. (21). Older individuals may also spend more time in outdoor activities such as gardening or visiting parks, which could further increase their exposure to grass and weed pollens (22).

### 4.4 Sex-specific patterns and age-by-sex interactions

Male patients had higher sensitization rates than females for most allergens. Multivariable models confirmed higher adjusted odds of sensitization to several inhalant allergens (mites, grasses, ragweed) and to milk in males, whereas females had higher odds of cat dander sensitization. These differences likely stem from a mix of exposure patterns and biological factors. For instance, boys may spend more time in active outdoor play and may be less shielded from indoor dust, increasing early exposure to mites and other allergens (6). Higher smoking rates among adult men could also influence IgE levels and airway inflammation (23).

We observed significant age-by-sex interactions for mite, egg white, and milk sensitization. In both sexes, sensitization to these allergens declined with age, but the decline was steeper in males. This suggests that boys may carry a particularly high burden of sensitization to key inhalant and food allergens in early life but lose it more rapidly over time, whereas sensitization in girls persists longer, particularly for food allergens. Several mechanisms may be at play. First, differential exposure patterns—for example, boys’ greater early-life contact with dust and outdoor environments—could drive more intense early sensitization and later tolerance (24). Second, sex-related differences in immune maturation and Th2-skewed responses may influence the persistence of sensitization; some evidence suggests that female immune systems maintain Th2-biased responses differently across developmental stages (25). Third, sex-specific dietary habits and health-seeking behaviors may modulate both exposure and recognition of food-related reactions (26–28). These explanations remain speculative without detailed exposure and hormonal data, and will need confirmation in prospective mechanistic studies.

### 4.5 Disease-specific sensitization profiles and clinical phenotypes

Looking at sensitization patterns by clinical phenotype, dust mites and milk stood out as core allergens across all seven disease categories, reflecting their high overall prevalence in this cohort. Among AR patients, mites and milk were the most common sensitizations, followed by mixed grass pollen and egg white. The profiles for CRS, asthma, urticaria, and allergic dermatitis were similar, with mites, milk, and mixed grasses at the top. AD was marked by high rates of sensitization to mites, milk, and egg white, while AC showed a different pattern, with mites, milk, and cat dander leading.

After adjusting for age and sex, multivariable models showed that disease-specific associations with individual allergens were generally modest compared with the strong effects of age and sex, though some remained significant. Urticaria was positively associated with egg white sensitization, and allergic dermatitis with mite sensitization—findings that support a link between certain skin phenotypes and food or indoor allergens (29). CRS, in contrast, showed no significant association with any of the seven major allergens we analyzed, suggesting that non-IgE-mediated mechanisms likely play an important role in many CRS cases (30).

We also observed a high comorbidity burden in AR patients: 25.9% had concurrent CRS and 6.3% had asthma, consistent with shared airway inflammation. The sensitization rate among AR patients was 60.6%—lower than the theoretical expectation of universal sensitization—which aligns with the concept of localized allergic rhinitis, where patients have IgE-mediated nasal reactivity to specific allergens despite negative systemic tests (31). This endotype may help explain some of the discordance between clinical symptoms and serum sIgE in our cohort.

Cat dander sensitization deserves particular attention. Females had higher adjusted odds of sensitization to cat dander, yet cat dander sensitization was negatively associated with several respiratory and ocular phenotypes (AR, asthma, AC). Rather than pointing to a protective effect, this pattern is more likely explained by reverse causation and behavioral avoidance: patients with established respiratory or ocular allergies are often advised to avoid cats, and many symptomatic individuals may choose not to own them. Because cat allergens such as Fel d 1 are highly adhesive and widely distributed (32), sensitization can still occur without direct pet ownership, while those who tolerate cats may be overrepresented among individuals with detectable cat dander sensitization. Without longitudinal exposure data, we cannot tease apart behavioral and biological mechanisms.

### 4.6 Annual variation and environmental context

Sensitization rates for most allergens varied significantly across the three study years, pointing to temporal fluctuations in this hospital-based population. These differences could stem from changes in environmental conditions (e.g., humidity, pollen seasons, air pollution), healthcare-seeking behavior, or the composition of patients referred for testing. Ningbo’s consistently high humidity and distinct spring and autumn pollen seasons likely help explain why mite and herbaceous pollen sensitization predominate (33). Future studies that combine clinical data with environmental monitoring—including aeroallergen counts, meteorological variables, and pollution indices—are needed to clarify what drives these temporal changes.

### 4.7 Clinical and public health implications

Our findings carry several implications for clinical practice. The clear age- and sex-specific sensitization patterns suggest that diagnostic panels and prevention strategies should be tailored accordingly. In preschool and school-age children, testing for milk, egg white, and mite sensitization is likely to yield the most useful information, whereas in older adults, pollen and mite sensitization become more relevant. The identification of mites and milk as “core” sensitizers across multiple phenotypes supports prioritizing environmental mite control and, where appropriate, dietary counseling.

Taken together, our results point to childhood as a critical window for intervention, with sex-specific considerations also warranting attention. The combination of higher early-life sensitization and a steeper age-related decline in boys calls for intensive environmental control and dietary counseling in early childhood, while the more persistent sensitization patterns in girls justify longer-term follow-up into adolescence, particularly for food sensitization. Incorporating these age- and sex-specific risk profiles into clinical decision-making could help allocate diagnostic testing and preventive resources more effectively.

### 4.8 Limitations

This study has several limitations. As a retrospective, single-center study of symptomatic patients referred for sIgE testing, our findings reflect hospital-based sensitization patterns in a selected population and cannot be generalized to estimate sensitization or allergy prevalence in the general population of Ningbo. sIgE positivity indicates sensitization rather than clinically relevant allergy, and we lacked systematic data on reaction timing, severity, skin prick tests, or provocation tests—particularly for foods—so we could not distinguish between silent sensitization and true clinical allergy. The allergen panel, while covering common inhalant and food allergens, may not include all regionally important allergens. In addition, although we used a standardized commercial platform for sIgE measurement, detailed performance characteristics (e.g., sensitivity, specificity, inter-assay variability) were not documented in patient records, so we cannot rule out the possibility that measurement variability introduced minor noise into the sensitization rates.

The cross-sectional, retrospective design also precludes causal inference. Apparent protective or risk associations—such as the negative association between cat dander sensitization and respiratory phenotypes—may be influenced by reverse causation and unmeasured confounding. Age-related trends may be influenced by cohort effects, changes in testing indications over time, and varying disease prevalence across age groups, rather than solely biological maturation. Finally, although we applied Benjamini–Hochberg false discovery rate control to account for multiple testing, the large number of comparisons across allergens, age groups, and disease categories means that some statistically significant findings could still reflect chance associations and should be interpreted with caution.

Despite these limitations, this study provides one of the largest hospital-based datasets on allergen sensitization in Ningbo and offers detailed age-, sex-, and phenotype-specific patterns that may inform local clinical practice and public health planning.

## 5 Summary

This large hospital-based epidemiological study examined allergen sensitization patterns among 19,787 patients with suspected allergic diseases in Ningbo, China, between 2019 and 2021, and yielded three main observations. First, allergen sensitization reflects a multifactorial interplay between genetic predisposition, environmental exposures, and age-related physiological changes, rather than being a static trait. Second, we identified distinct developmental trajectories: sensitization to food allergens—particularly milk and egg white—peaked in early childhood and declined thereafter, whereas sensitization to plant-derived aeroallergens, such as grass and weed pollens, became more prominent in adulthood. Third, sensitization profiles varied by clinical phenotype: dust mites and milk emerged as core sensitizers shared across multiple disease categories, while other allergens, such as cat dander, showed more phenotype-specific patterns.

These findings underscore the clinical necessity of region- and age-specific allergen screening strategies. Precise identification of prevalent sensitizers in Ningbo may facilitate: (1) development of diagnostic algorithms tailored to local sensitization profiles; (2) implementation of individualized avoidance and environmental control measures; and (3) refinement of patient and public education initiatives. Integrating these epidemiological insights into precision medicine and secondary prevention frameworks could help reduce the burden of allergic diseases in Eastern China, while acknowledging that sIgE positivity reflects sensitization and does not by itself confirm clinical allergy.

These findings highlight the need for region- and age-specific allergen screening strategies in clinical practice. Accurate identification of prevalent sensitizers in Ningbo can support several goals: developing diagnostic algorithms tailored to local sensitization profiles, implementing individualized avoidance and environmental control measures, and refining patient and public education initiatives. Integrating these epidemiological insights into precision medicine and secondary prevention frameworks could help reduce the burden of allergic diseases in eastern China, while keeping in mind that sIgE positivity reflects sensitization and does not by itself confirm clinical allergy.

## Data Availability

The raw data supporting the conclusions of this article will be made available by the authors, without undue reservation.
