# Supplementary material for: Hospital-based patterns of allergen sensitization among 19,787 patients with suspected allergic diseases in Ningbo, China
Source: Front Public Health. 2026 Apr 24;14:1786052. doi: 10.3389/fpubh.2026.1786052 (PMC13152857; doi:10.3389/fpubh.2026.1786052)
Supplement: Supplementary file 1 [file Data_Sheet_1.DOCX]

Supplementary Material

# Supplementary Figures and Tables

## Supplementary Tables

| **Table S1. Multivariate logistic regression analysis and age trend tests for sensitization to seven common allergens** | | | | | | | |
| --- | --- | --- | --- | --- | --- | --- | --- |
| Variable | Ragweed(n=19786) | Peanuts(n=19786) | Dust mites(n=19786) | Mixed grass(n=19786) | Egg white(n=19786) | Cat dancer(n=19786) | Milk(n=19786) |
|  | OR(95%CI) | OR(95%CI) | OR(95%CI) | OR(95%CI) | OR(95%CI) | OR(95%CI) | OR(95%CI) |
| Age (per 10 years) | 1.069(1.006-1.135)* | 0.927(0.876-0.982)** | 0.759(0.740-0.778)*** | - | - | - | - |
| Age group (ref:18-35 years) |  |  |  |  |  |  |  |
| 2-5 years | - | - | - | 1.199(0.509-2.828) | 5.615(2.984-10.569)*** | 0.412(0.099-1.703) | 4.183(2.591-6.755)*** |
| 6-13 years | - | - | - | 0.538(0.403-0.718)*** | 4.596(3.693-5.719)*** | 0.664(0.515-0.857)** | 3.700(3.234-4.233)*** |
| 14-17 years | - | - | - | 0.551(0.353-0.861)** | 1.840(1.275-2.653)* | 1.071(0.773-1.486) | 2.511(2.066-3.053)*** |
| 36-59 years | - | - | - | 1.501(1.264-1.782)*** | 0.880(0.690-1.123) | 0.323(0.256-0.408)*** | 0.380(0.322-0.447)*** |
| >60 years | - | - | - | 1.393(1.056-1.837)* | 0.835(0.547-1.274) | 0.130(0.072-0.236)*** | 0.397(0.293-0.539)*** |
| P for trend |  |  |  | <0.001 | <0.001 | <0.001 | <0.001 |
| Age × Sex interaction  (P value) | 0.489 | 0.672 | 0.012 | 0.361 | 0.016 | 0.43 | <0.001 |
| Female vs. Male | 0.742(0.606-0.909)** | 1.142(0.948-1.374) | 0.778(0.721-0.840)*** | 0.677(0.581-0.788)*** | 1.130(0.951-1.341) | 1.437(1.197-1.724)*** | 0.872(0.780-0.975)* |
| allergic rhinitis | 1.433(0.723-2.842) | 0.715(0.333-1.535) | 0.750(0.563-1.000)* | 1.182(0.838-1.668) | 1.378(0.933-2.035) | 0.388(0.220-0.685)* | 1.028(0.715-1.479) |
| chronic rhinosinusitis | 1.748(0.841-3.633) | 0.776(0.351-1.716) | 1.187(0.879-1.603) | 1.244(0.848-1.826) | 1.391(0.900-2.149) | 0.616(0.332-1.146) | 1.194(0.810-1.760) |
| asthma | 1.214(0.572-2.574) | 0.888(0.382-2.065) | 0.716(0.521-0.983)* | 1.019(0.682-1.525) | 1.377(0.818-2.317) | 0.204(0.110-0.378)*** | 1.364(0.865-2.153) |
| urticaria | 1.135(0.563-2.287) | 0.945(0.431-2.075) | 1.250(0.931-1.680) | 0.960(0.671-1.374) | 1.669(1.093-2.547)* | 0.821(0.449-1.501) | 1.060(0.725-1.549) |
| atopic dermatitis | 1.015(0.426-2.420) | 0.406(0.173-0.954)* | 0.955(0.665-1.371) | 0.651(0.385-1.100) | 0.999(0.592-1.684) | 0.468(0.217-1.006) | 1.033(0.657-1.622) |
| allergic dermatitis | 1.277(0.570-2.861) | 0.707(0.301-1.659) | 1.536(1.094-2.156)* | - | - | - | - |
| atopic conjunctivitis | - | - | - | 1.351(0.641-2.848) | 1.724(0.854-3.480) | 0.241(0.115-0.505)*** | 0.962(0.615-1.504) |

*p < 0.05, **p < 0.01, ***p < 0.001.

| **Table S2. Sex-stratified age effects for allergens with significant age-by-sex interaction.** | | | |
| --- | --- | --- | --- |
| Allergen Species | Age metric | Males OR (95% CI) | Females OR (95% CI) |
| Dust mite | Per 10 years | 0.969(0.966-0.973)*** | 0.976(0.972-0.979)*** |
| Egg white | Per unit age score | 0.637(0.584-0.695)*** | 0.696(0.642-0.755)*** |
| Milk | Per unit age score | 0.679(0.646-0.714)*** | 0.757(0.717-0.799)*** |

*p < 0.05, **p < 0.01, ***p < 0.001.

## Supplementary Figures


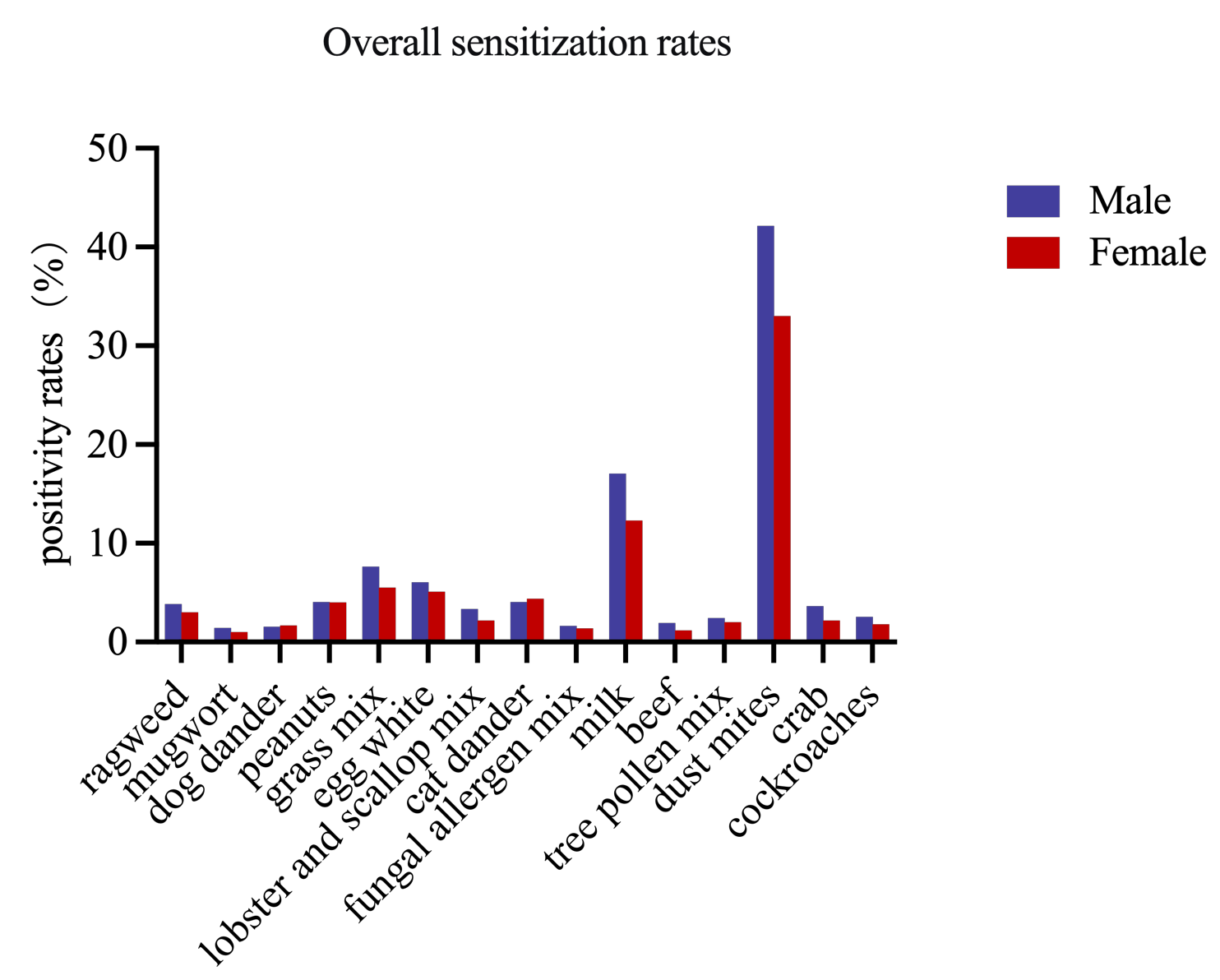


**Supplementary Figure 1.** Sensitization Rates of Allergen Species in Sex

1.
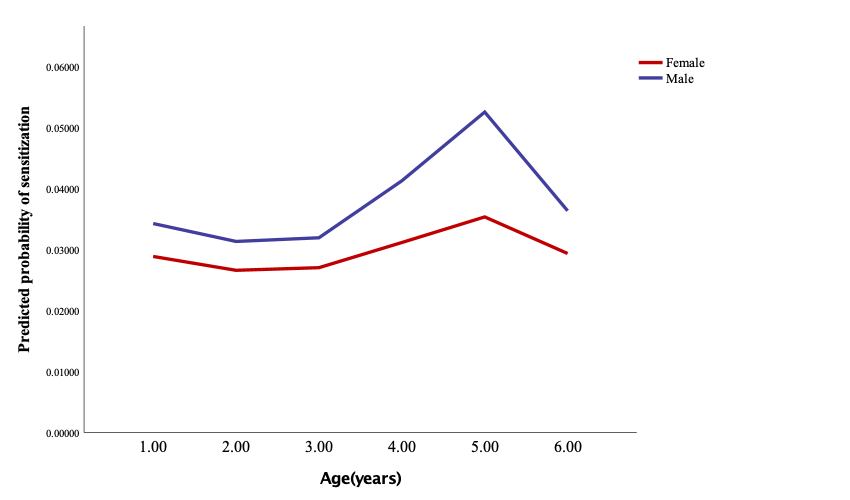

2.
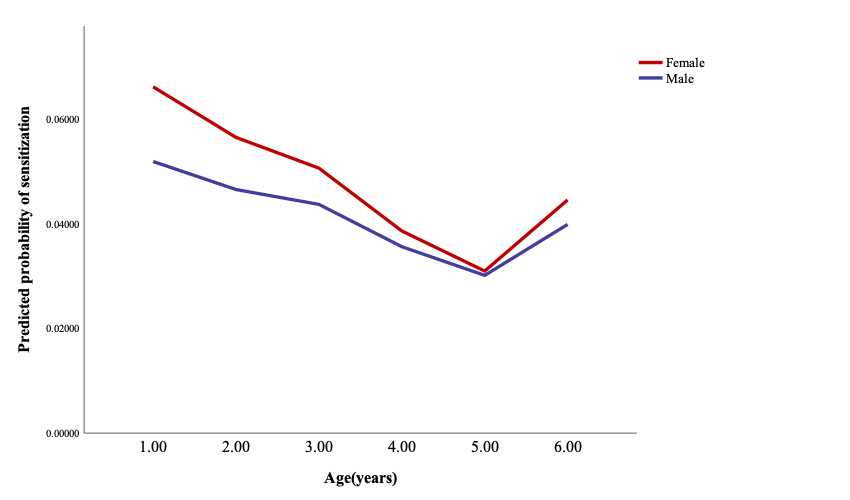

3.
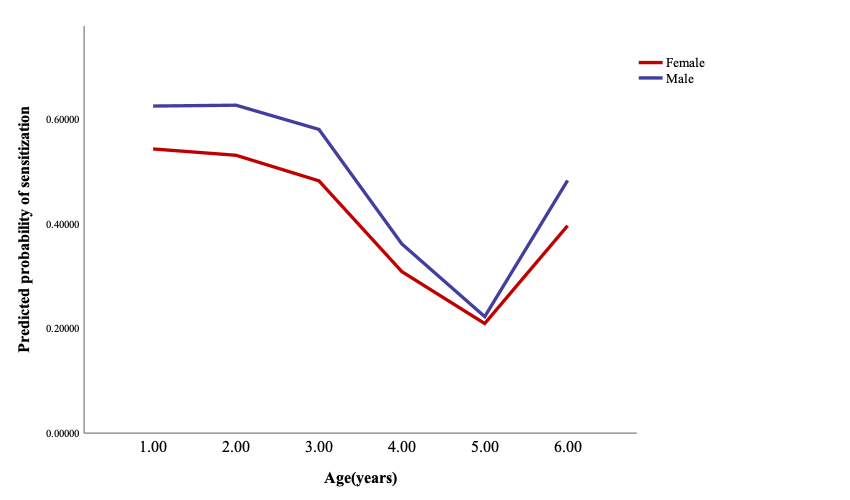

4.
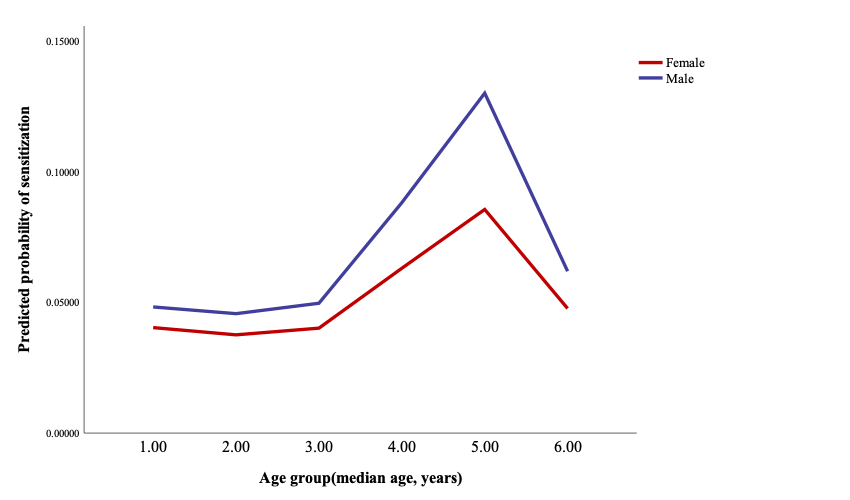

5.
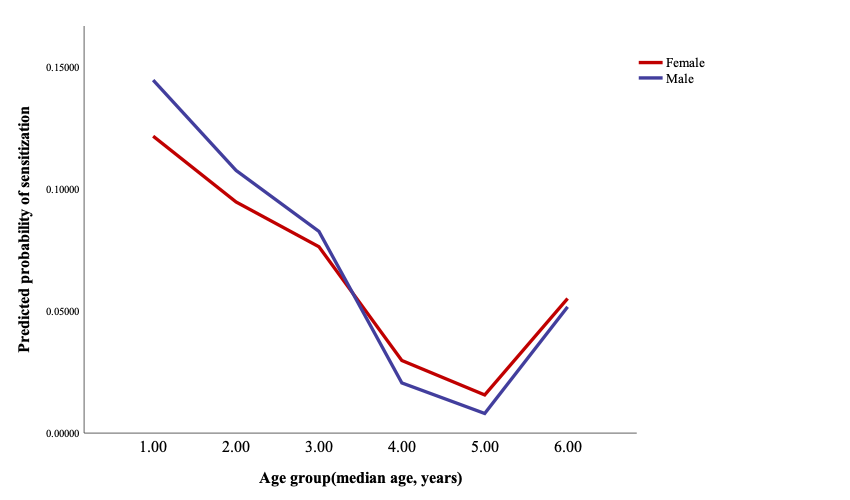

6.
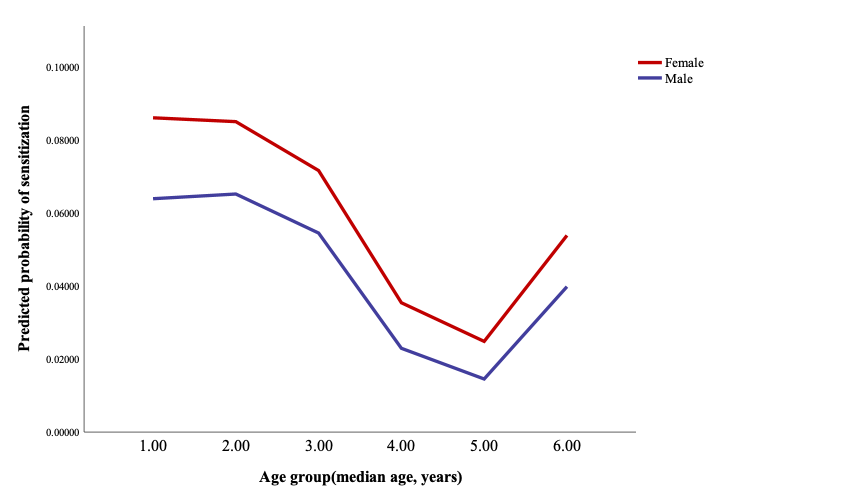

7.
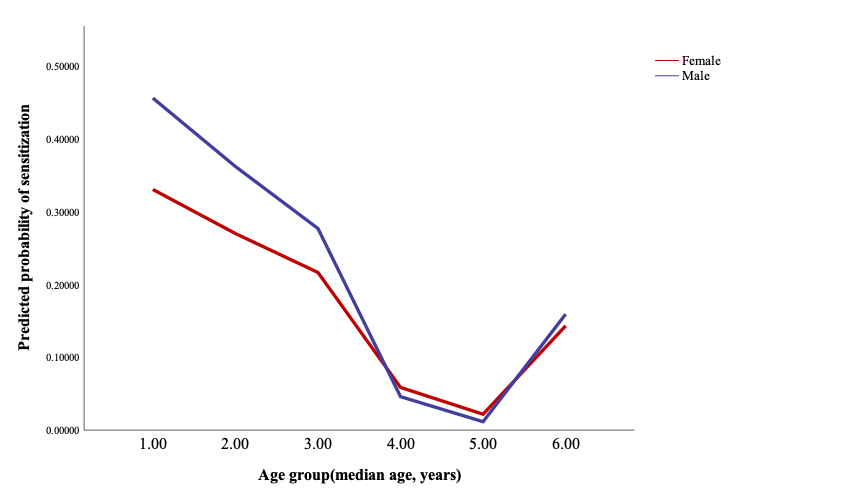


**Supplementary Figure 2.** Age trends in predicted probability of sensitization to seven common allergens by sex. (A) ragweed; (B) peanuts; (C) dust mites; (D) mixed grass; (E) egg white; (F) cat dancer; (G) milk. Predicted probabilities were derived from multivariate logistic regression models adjusted for allergic rhinitis, chronic rhinosinusitis, asthma, urticaria, atopic dermatitis, allergic dermatitis, and atopic conjunctivitis. Significant age-by-sex interactions were observed for dust mite (C), egg white (E), and milk (G) (all P for interaction < 0.05).
